# Murphy’s Parturition

**Published:** 1847-08

**Authors:** 


### MURPHY'S PARTURITION.

We have received from the publishers, at New York, Messrs. S. & W. Wood, to whom we are indebted for the work of Dr. Beck just referred to, a copy of the admirable Lectures of Prof. Murphy, of the London University, on "Natural and Difficult Parturition."

At present we have only room to speak of the volume in general terms, but we shall avail ourselves of the earliest opportunity to notice it at the length to which its merits entitle it. The many favorable notices of the work by English critics which we have seen, induced a strong desire to see an early edition of it brought before the American profession. That desire has been fulfilled, and we have no doubt the work will become as popular in this country as the author is himself, as a practitioner and gentleman, at home.

